# Diagnostic and Prognostic Utility of the Extracellular Vesicles Subpopulations Present in Pleural Effusion

**DOI:** 10.3390/biom11111606

**Published:** 2021-10-29

**Authors:** Joman Javadi, André Görgens, Hanna Vanky, Dhanu Gupta, Anders Hjerpe, Samir EL-Andaloussi, Daniel Hagey, Katalin Dobra

**Affiliations:** 1 Division of Pathology, Department of Laboratory Medicine, Karolinska Institutet, 141 52 Stockholm, Sweden; hanna.hjerpe.vanky@stud.ki.se (H.V.); anders.hjerpe@ki.se (A.H.); katalin.dobra@ki.se (K.D.); 2 Division of BCM, Department of Laboratory Medicine, Karolinska Institutet, 141 52 Stockholm, Sweden; andre.gorgens@ki.se (A.G.); dhanu.gupta@ki.se (D.G.); Samir.el-andaloussi@ki.se (S.E.-A.); daniel.hagey@ki.se (D.H.)

**Keywords:** malignant pleural mesothelioma, pleural effusion, extracellular vesicles, biomarkers

## Abstract

Extracellular vesicles (EVs), comprising exosomes, microvesicles, and apoptotic bodies, are released by all cells into the extracellular matrix and body fluids, where they play important roles in intercellular communication and matrix remodeling in various pathological conditions. Malignant pleural mesothelioma (MPM) is a primary tumor of mesothelial origin, predominantly related to asbestos exposure. The detection of MPM at an early stage and distinguishing it from benign conditions and metastatic adenocarcinomas (AD) is sometimes challenging. Pleural effusion is often the first available biological material and an ideal source for characterizing diagnostic and prognostic factors. Specific proteins have previously been identified as diagnostic markers in effusion, but it is not currently known whether these are associated with vesicles or released in soluble form. Here, we study and characterize tumor heterogeneity and extracellular vesicle diversity in pleural effusion as diagnostic or prognostic markers for MPM. We analyzed extracellular vesicles and soluble proteins from 27 pleural effusions, which were collected and processed at the department of pathology and cytology at Karolinska University Hospital, representing three different patient groups, MPM (*n* = 9), benign (*n* = 6), and AD (*n* = 12). The vesicles were fractionated into apoptotic bodies, microvesicles, and exosomes by differential centrifugation and characterized by nanoparticle tracking analysis and Western blotting. Multiplex bead-based flow cytometry analysis showed that exosomal markers were expressed differently on EVs present in different fractions. Further characterization of exosomes by a multiplex immunoassay (Luminex) showed that all soluble proteins studied were also present in exosomes, though the ratio of protein concentration present in supernatant versus exosomes varied. The proportion of Angiopoietin-1 present in exosomes was generally higher in benign compared to malignant samples. The corresponding ratios of Mesothelin, Galectin-1, Osteopontin, and VEGF were higher in MPM effusions compared to those in the benign group. These findings demonstrate that relevant diagnostic markers can be recovered from exosomes.

## 1. Introduction

Malignant pleural mesothelioma (MPM) is an aggressive mesenchymal tumor arising from mesothelial cells of pleura, characterized by the production of hyaluronan and a spectrum of cell surface and matrix proteoglycans, some of which are useful biomarkers facilitating early clinical diagnosis, the monitoring of tumor burden, and effect of therapy. Early diagnosis from pleural effusion is challenging but essential to improving patient survival [1,2]. As pleural effusion is the first available clinical material, cytological examination provides the earliest diagnostic evaluation [3,4] by combining cytomorphology, immunocytochemistry, fluorescence in situ hybridization, biomarker analyses, and electron microscopy [3,5,6]. In addition to early diagnosis, distinguishing MPM from metastatic adenocarcinoma (AD) is another challenge in patients with malignant pleural effusion. CEA, HBME1, TTF1, CK7, and Calretinin are the most useful immunohistochemistry markers for AD [3,7]. 

The tumor tissue microenvironment is an important and dynamic regulator of tumor progression and development of metastases [8]. This is also true for the malignant effusion, which contains various soluble factors facilitating or inhibiting this process. Cells communicate with their surroundings, thereby regulating the behavior of benign cells and the extracellular matrix [9]. The secretion of factors for paracrine stimulation is one way to achieve such regulation, while the formation extracellular vesicles is another way to transfer signals to neighboring cells [10].

Extracellular vesicles (EVs), comprising exosomes, microvesicles, and apoptotic bodies, are heterogeneous nanoparticles ranging from 30 nm to 4 μm. They are released into the extracellular matrix and body fluids by all cell types but arise via distinct biological processes. For instance, apoptotic bodies are the largest (1–4 μm), as they are formed during programmed cell death. In contrast, all cells release exosomes, which are the smallest (50–100 nm) and formed during the maturation of endosomes into multi-vesicular bodies by inward vesiculation of the endosomal membrane, and microvesicles, which are between 200 nm and 1 μm and formed by outward budding of the plasma membrane [8,11,12,13,14,15,16]. Although there are no definitive markers to completely separate these vesicle populations, apoptotic bodies are known to contain apoptotic material, such as cleaved caspase 9. It is currently impossible to separate microvesicles and exosomes. However, microvesicles are enriched in the cell surface tetraspanin CD9, while exosomes are enriched in CD81 [17,18,19].

The released EVs carry functional factors, such as lipids, proteins, and nucleic acids, to recipient cells, which highlights their importance as mediators of cell-to-cell communication [15,20,21]. Vesicles secreted into the extracellular fluid can be taken up by target cells via direct fusion with the plasma membrane or by endocytosis. This transfer of their bioactive content can then regulate intracellular signaling pathways or gene expression in the recipient cells [22]. On the other hand, this EV cargo can also be utilized a biomarker of disease [23].

Pleural effusions contain different cells, including macrophages, lymphocytes, neutrophils, mesothelial cells, as well as, in malignant conditions, tumor cells of various origin [24,25]. All these cells may secrete EVs into the pleura, and bear heterogenous molecular surface markers, such as CD81, CD63, and CD9, proteins, RNAs, or DNA, which can be used as biomarkers [26,27,28]. We have previously shown the diagnostic applicability of a series of biomarkers and optimized a Luminex based multiparameter battery to assess them [29]. Some of these factors are biologically active, regulating cell growth and angiogenesis. Their appearance in the effusion fluid may reflect the tumor promoting effects of the tumor cells themselves or the reaction of the surrounding benign tissues. Since these factors are potential targets for individualized treatment, it is interesting to understand how the expression of such factors varies from case to case and if the factors are transported in association with different vesicles or released directly into the effusion supernatant. 

The aim of the present investigation is to study the nature and variability of these factors in effusion caused by adenocarcinoma, malignant mesothelioma, and benign reactivity. To achieve this, we have used differential centrifugation to separate the cells and different classes of EVs from the soluble components within pleural effusion. We then analyze these components using Western blotting, nanoparticle tracking (NTA), multiplex bead-based flow cytometric EV surface protein profiling, and Luminex antigen detection. A better understanding of the factors related to vesicle mediated intercellular communication may give us tools to monitor cancer progression and identify novel therapeutic targets.

## 2. Materials and Methods

### 2.1. Sample Collection and Study Design

Pleural effusions from patients with malignant pleural mesothelioma (MPM; *n* = 9), metastatic lung adenocarcinoma (AD; *n* = 12), and benign reactive mesothelial proliferations (BE; *n* = 6) were evaluated. Pleural effusions were collected at different time point at the Department of Pathology and Cytology, Karolinska University Hospital by thoracocentesis under ethical permit number 2009/1138-31/3. After initial centrifugation, the cell pellets were taken for diagnostic cytology (Figure S1) and clinically established biomarker analysis, while the remaining effusions were kept at 4 °C until processing. 

### 2.2. Fractionation of Extracellular Vesicles

**Figure 1:**
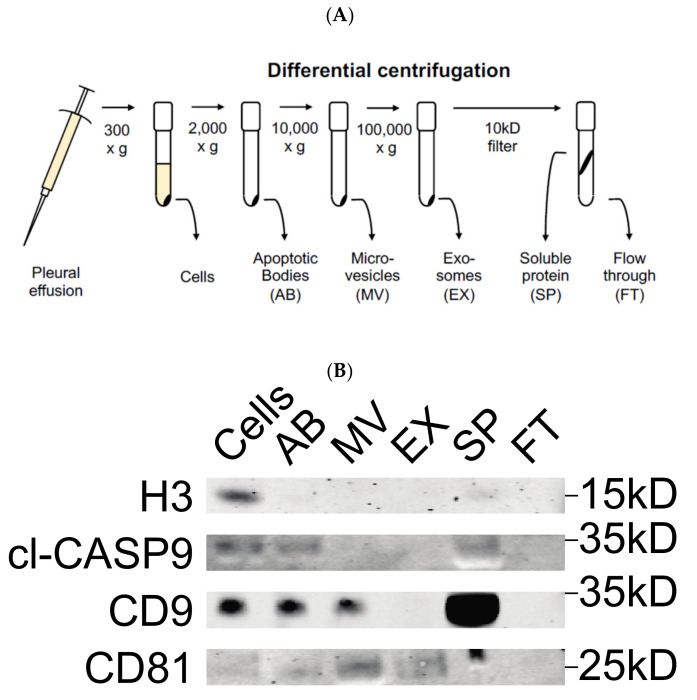
(**A**) Schematic of differential centrifugation protocol. (**B**) Western blot analysis of differential centrifugation fractions. The abundance of common markers (Histone H3 (H3), cleaved caspase-9 (cl-CASP9), CD9 and CD81) in different fractions was determined using Western blot. The molecular weight of each protein is shown on the right.

All samples were centrifuged at 300× *g* for 10 min to collect any remaining cells. Supernatants were centrifuged further at 2000× *g* for 10 min to isolate apoptotic bodies (ABs) and 10,000× *g* for 10 min to isolate microvesicles. Further centrifugation at 100,000× *g* for 90 min sedimented exosomes, while dissolved free proteins remained in the supernatant. The exosome pellets were resuspended in filtered PBS and kept at −80 °C for further analysis. All ultracentrifugation steps were performed using the Beckman Coulter (Brea, CA, USA) Type 70 Ti rotor at 4 °C. Furthermore, supernatant free soluble proteins were concentrated by centrifugal filters (Amicon Ultra-15, REF UFC901024) (Figure 1A).

### 2.3. NTA Analysis

To ascertain the average concentrations and sizes of exosomes in the samples, nanoparticle tracking analysis (NanoSight Techniques, LM, Malvern, UK) was used according to manufacturer’s instructions characterizing nanoparticles from 10–1000 nm in solution. The exosomes were diluted in PBS (1:2500) and applied directly to the NanoSight LM 10.

### 2.4. Luminex Assay with Human Premixed Multi-Analyte Kit

Two human premixed multi-analyte kits from R&D system were used to assess the levels of 10 different biomarkers in exosomes and soluble protein derived from pleural effusion. The first kit (cat: LXSAHM-09, Minneapolis, MN, USA) was used for analyzing Angiopoietin-1, HGF, MMP-7, Osteopontin, TIMP-1, Galectin, Mesothelin, NRG1-b1, and Syndecan-1 simultaneously. The second kit (cat: LXSAHM-01, Minneapolis, MN, USA) was used for analyzing VEGF. In total, we analyzed 27 pleural effusions, of which 9 were from MPM patients, 12 from AD patients, and 6 were benign effusions. Effusions were diluted 5-fold using the dilution buffer included in the kit. All standards and samples were assayed in duplicate according to the manufacturer’s instructions.

### 2.5. Multiplex Bead-Based EV Flow Cytometry Assay

Different fractions of pleural effusion from MPM, AD, and BE patients (centrifuged at 2000× *g*, 10,000× *g*, and 120,000× *g*) were analyzed by bead-based multiplex EV flow cytometry assays (MACSPlex Exosome Kit, human, Miltenyi Biotec Corston, UK), following the manufacturer’s instructions with slight modifications [30]. The MACSPlex Exosome Kit allows detection of 37 surface epitopes (CD1c, CD2, CD3, CD4, CD8, CD9, CD11c, CD14, CD19, CD20, CD24, CD25, CD29, CD31, CD40, CD41b, CD42a, CD44, CD45, CD49e, CD56, CD62P, CD63, CD69, CD81, CD86, CD105, CD133/1, CD142, CD146, CD209, CD326, HLA-ABC, HLA-DRDPDQ, MCSP, ROR1, and SSEA-4), plus two internal isotype controls (mIgG1 and REA). Briefly, samples were incubated with the antibody coated MACSPlex Exosome Capture Beads. Subsequently, EVs bound to the MACSPlex Exosome Capture Beads were labeled with the MACSPlex Pan-Exosome Detection Reagents (APC-conjugated antiCD9, anti-CD63, and anti-CD81 detection antibody). Consequently, these complexes were analyzed by flow cytometry (MACSPlex Analyzer 10 Bergisch Gladbach, Germany) based on the fluorescence characteristics of both beads and the detection reagent, as described previously [30]. Positive signals indicate the abundance of the respective surface epitope on EVs within the sample.

### 2.6. Western Blot Analysis

To verify the content of the differential centrifugation fractions, Western blotting was performed with antibodies directed against well described cellular components. Albumin and IgG were removed using SpinTrap columns (28-9480-20, GE Healthcare, Buckinghamshire, UK) according to the manufacturer’s instructions. Sample fractions were prepared in 0.5M DTT, 8% SDS, 0.4 M Sodium carbonate, and 10% Glycerol and boiled for 5 min at 95 °C. Then, 20 µL of the samples were loaded on a NuPAGE Novex 4–12% (*w*/*v*) Bis-Tris pre-cast SDS-PAGE gel (NP0321BOX, Invitrogen, New York, NY, USA). Gels were run for 90 min at 120 V. Gels were transferred to a nitrocellulose membrane using the iBlot system (LC2009, Invitrogen, New York, NY, USA) according to the manufacturer’s instructions and then incubated at room temperature for 60 min in blocking buffer (927-70001, Li-Cor, Lincoln, NE, USA). After this point, the membrane was incubated overnight at 4 °C with primary anti-bodies in 1:1 blocking buffer: PBST. We used the following antibodies: CD9 (Abcam ab-92726, 1:2000, Cambridge, UK), cleaved-caspase 9 (Cell Signaling 9505S, 1:1000, Stockholm, Sweden), CD81 (Santa Cruz sc-9158, 1:200, Danvers, MA, USA), and histone H3 (Santa Cruz FL-136, 1:250, Danvers, MA, USA). After washing 3 × 15 min in PBST, freshly prepared secondary antibody in PBST (Li-Cor 926-68020, 926-152 68071, 926-68079, 1:15,000) was added to the membrane and incubated for 60 min at room temperature followed by 3 × 15 min washes with PBST. Membranes were developed using both 700- and 800-nm channels on the LI-COR Odyssey Imager and exported from the Li-Cor Image Studio 5.2 software.

## 3. Results

In order to comprehensively separate the vesicles and soluble proteins associated with BE, MPM, and AD pleural effusion, we collected samples from 27 patients and subjected them to differential centrifugation [31]. Due to the distinct sizes and densities of these components, this allowed us to separate the cells, apoptotic bodies, microvesicles, and exosomes from the soluble proteins within the effusion.

### 3.1. Validation of Differential Centrifugation Fraction of Pleural Effusion

To verify that the fractions isolated from pleural effusions contained the relevant hypothesized components, we performed Western blot against proteins known to be enriched in each. Although we detected Histone H3 specifically in the cell fraction, cleaved Caspase-9 was found in cells, apoptotic bodies, and soluble protein. As a primarily cell surface tetraspanin, CD9 was detected in cells, apoptotic bodies, microvesicles, and soluble protein. In contrast, CD81 was primarily found in association with the microvesicle and exosome fractions (Figure 1B). These results validated that differential centrifugation was successful in separating the hypothesized components within pleural effusion.

### 3.2. Measurement of Exosome Particle Size and Concentration

**Figure 2:**
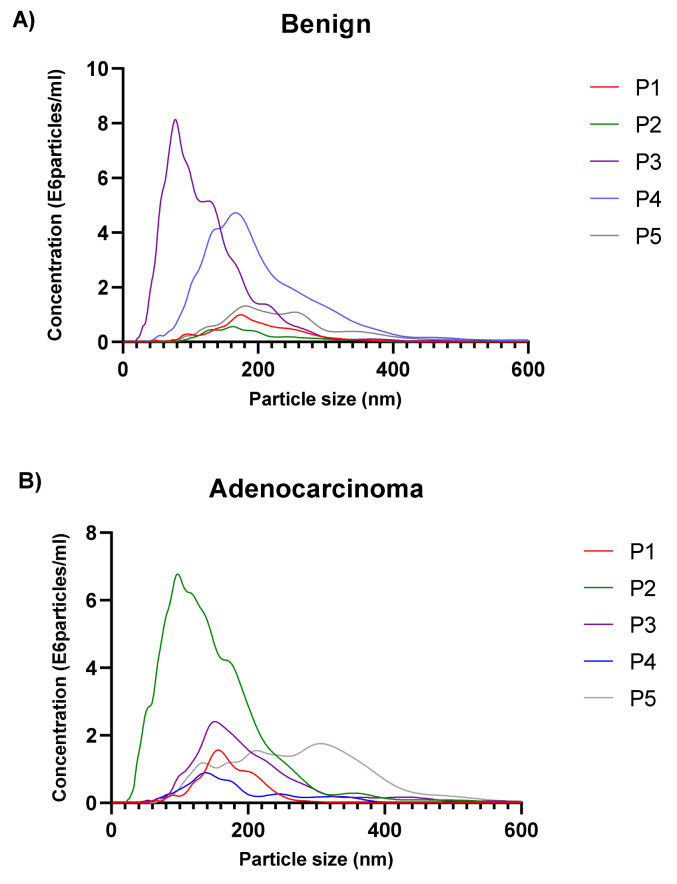

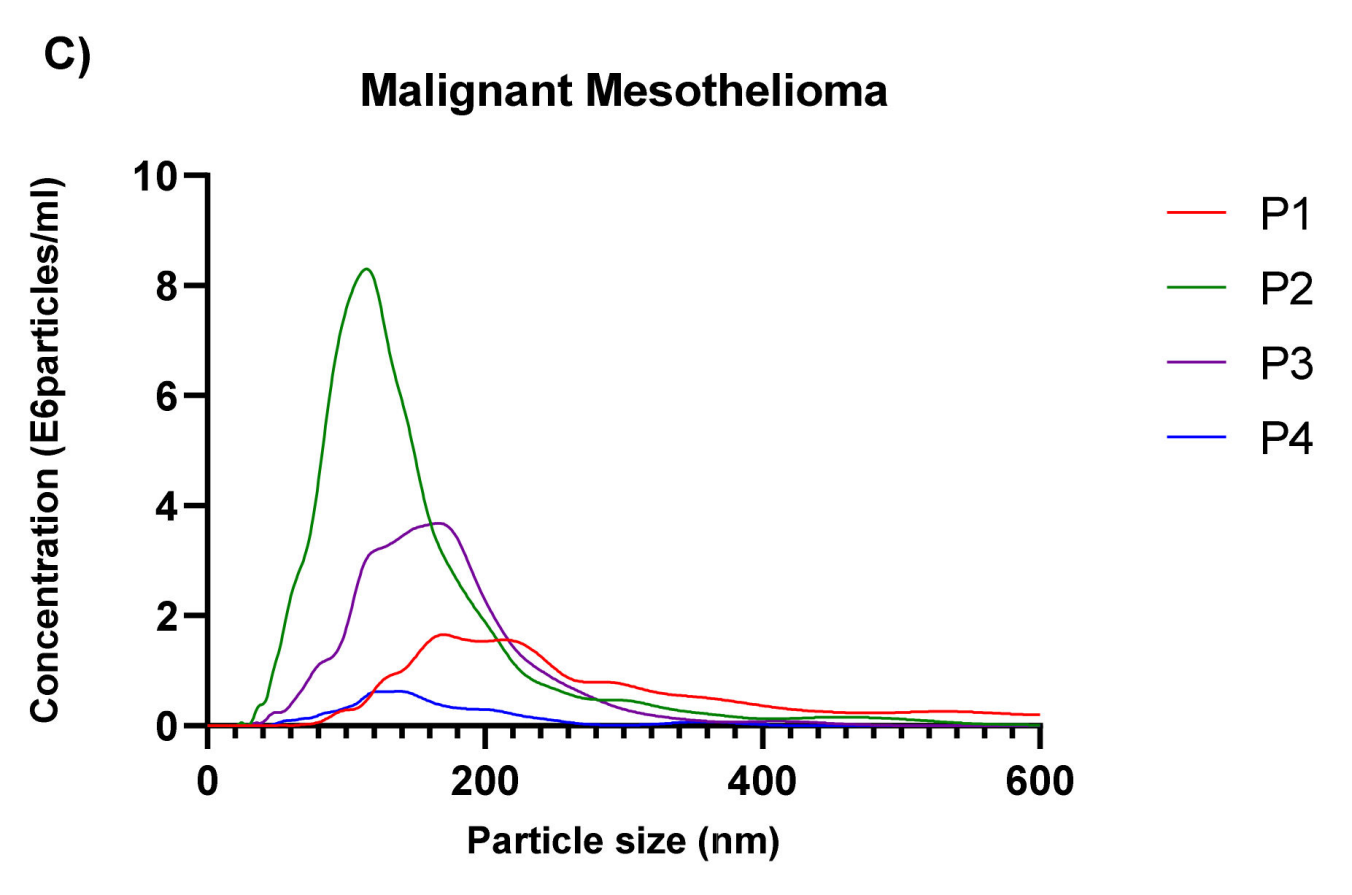
Exosome’s size and concentration measured by NTA. Size distribution plots from five benign pleural effusions (0.7 × 10^6^ to 8.7 × 10^6^ particles/mL) (**A**), five adenocarcinoma patients (0.7 × 10^6^ to 7.4 × 10^6^ particles/mL) (**B**) and four malignant mesothelioma patients (0.6 × 10^6^ to 8.7 × 10^6^ particles/mL) (**C**).

The concentration and size distribution of particles in the exosome fraction were assessed by NTA in 14 patients: five benign, five AD, and four MPM. The size distribution ranged from 30 to 600 nm in diameter and the vesicular concentration varied between individuals (0.6 × 10^6^ to 8.74 × 10^6^ particles/mL) (Figure 2A–C). The mean exosome concentration was 3.8 × 10^6^, 3.1 × 10^6^, and 3.5 × 10^6^ particles/mL for MPM, AD, and benign patients, respectively.

### 3.3. Detection of Surface Protein Markers

**Figure 3:**
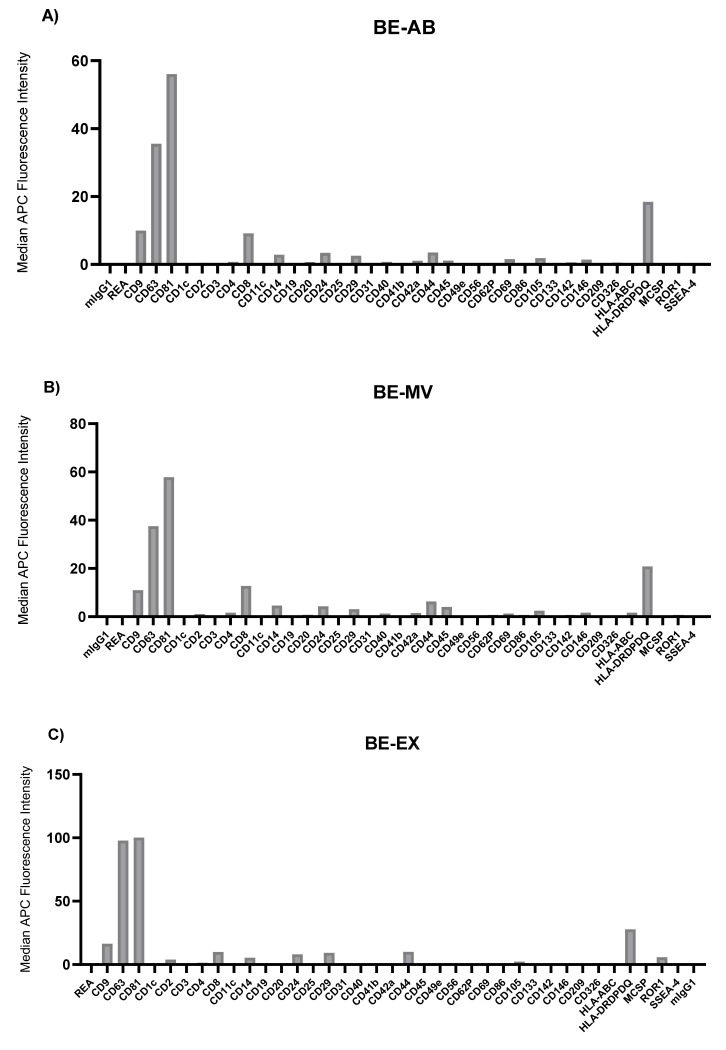

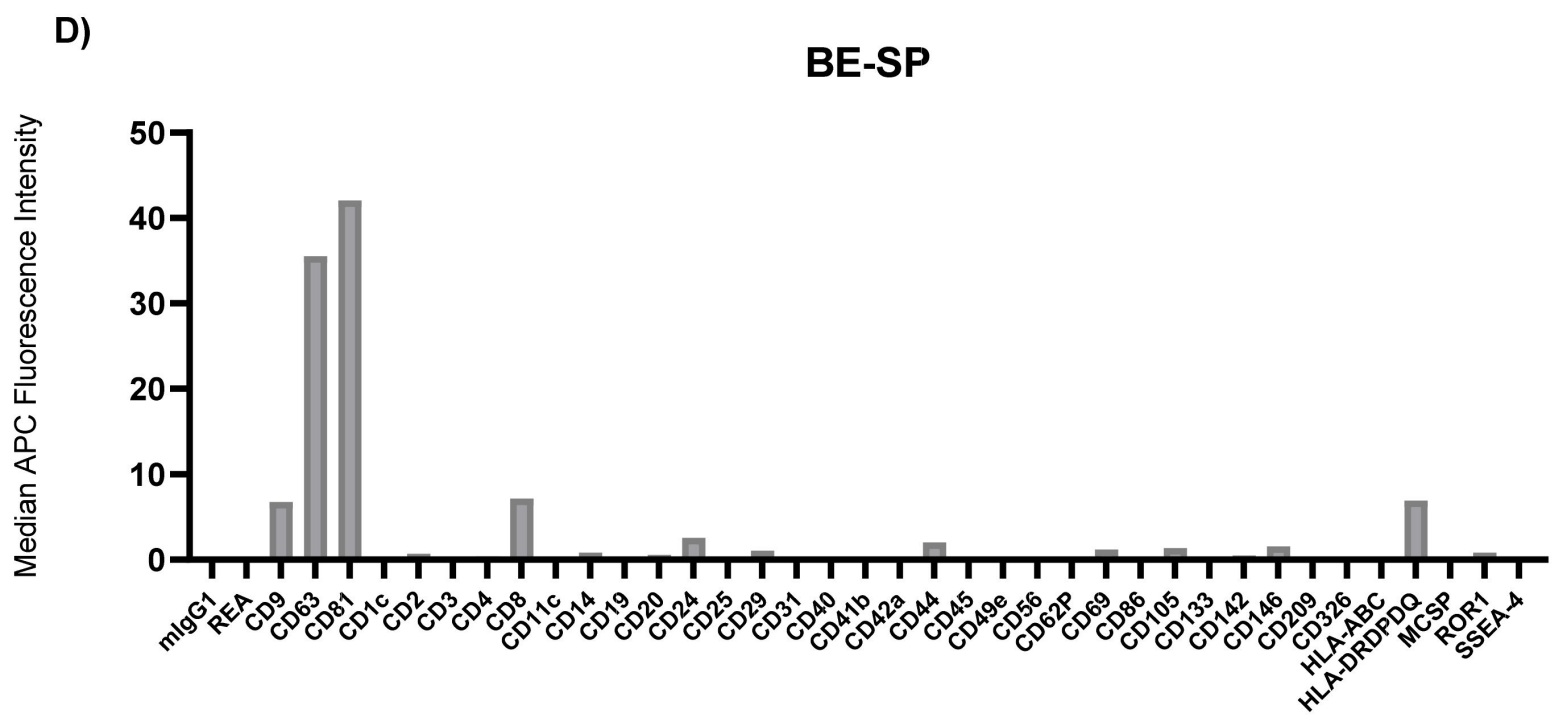
Median fluorescence intensity EV surface protein profiles of benign (BE) pleural effusion differential centrifugation fractions: apoptotic bodies (AB) (**A**), microvesicles (MV) (**B**), exosomes (EX) (**C**) and soluble protein in supernatant (SP) (**D**). mIgG1 and REA indicate isotype control and represent negative markers. Each bar corresponds to one surface epitope measurement, as indicated on the x-axis.

**Figure 4:**
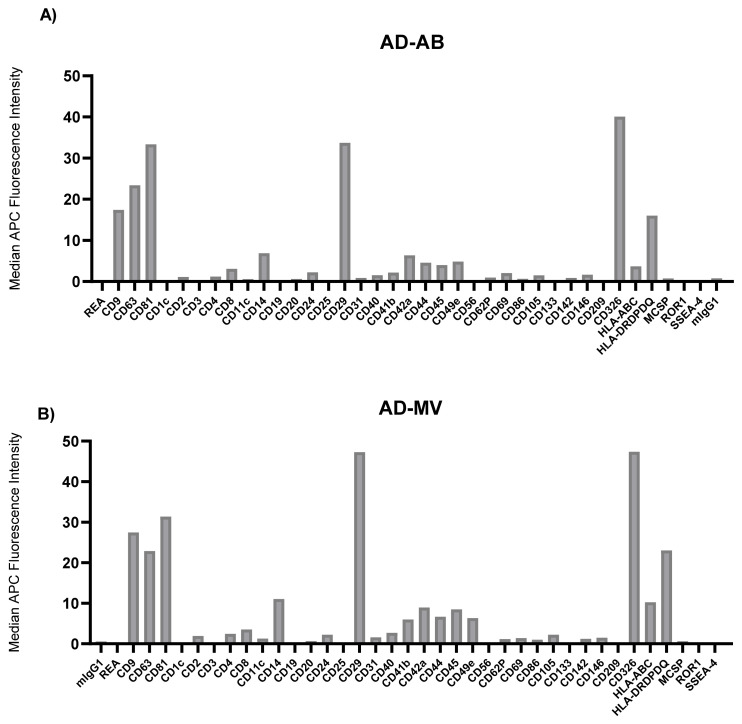

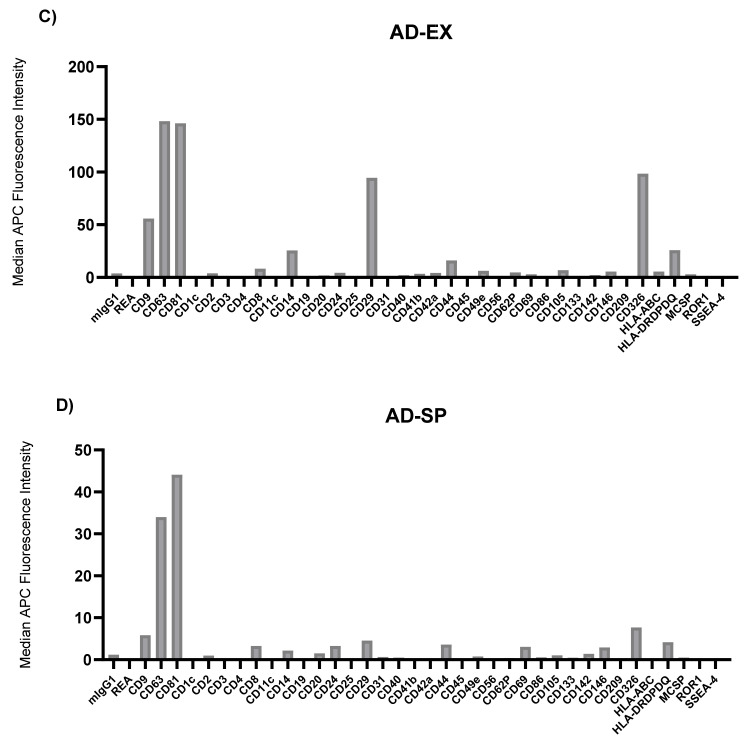
Median fluorescence intensity EV surface protein profiles of adenocarcinoma (AD) pleural effusion differential centrifugation fractions: apoptotic bodies (AB) (**A**), microvesicles (MV) (**B**), exosomes (EX) (**C**) and soluble protein in supernatant (SP) (**D**). mIgG1 and REA indicate isotype control and represent negative markers. Each bar corresponds to one surface epitope measurement, as indicated on the x-axis.

**Figure 5:**
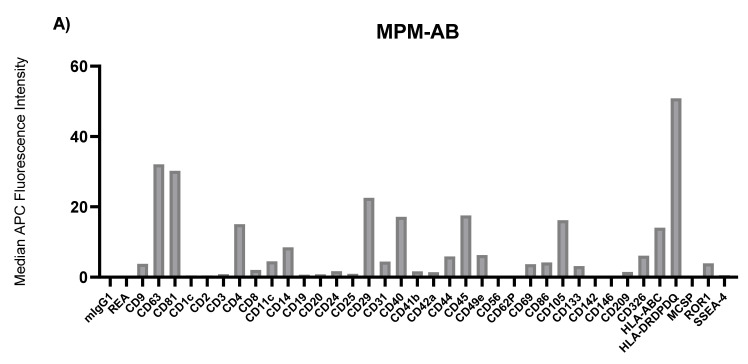

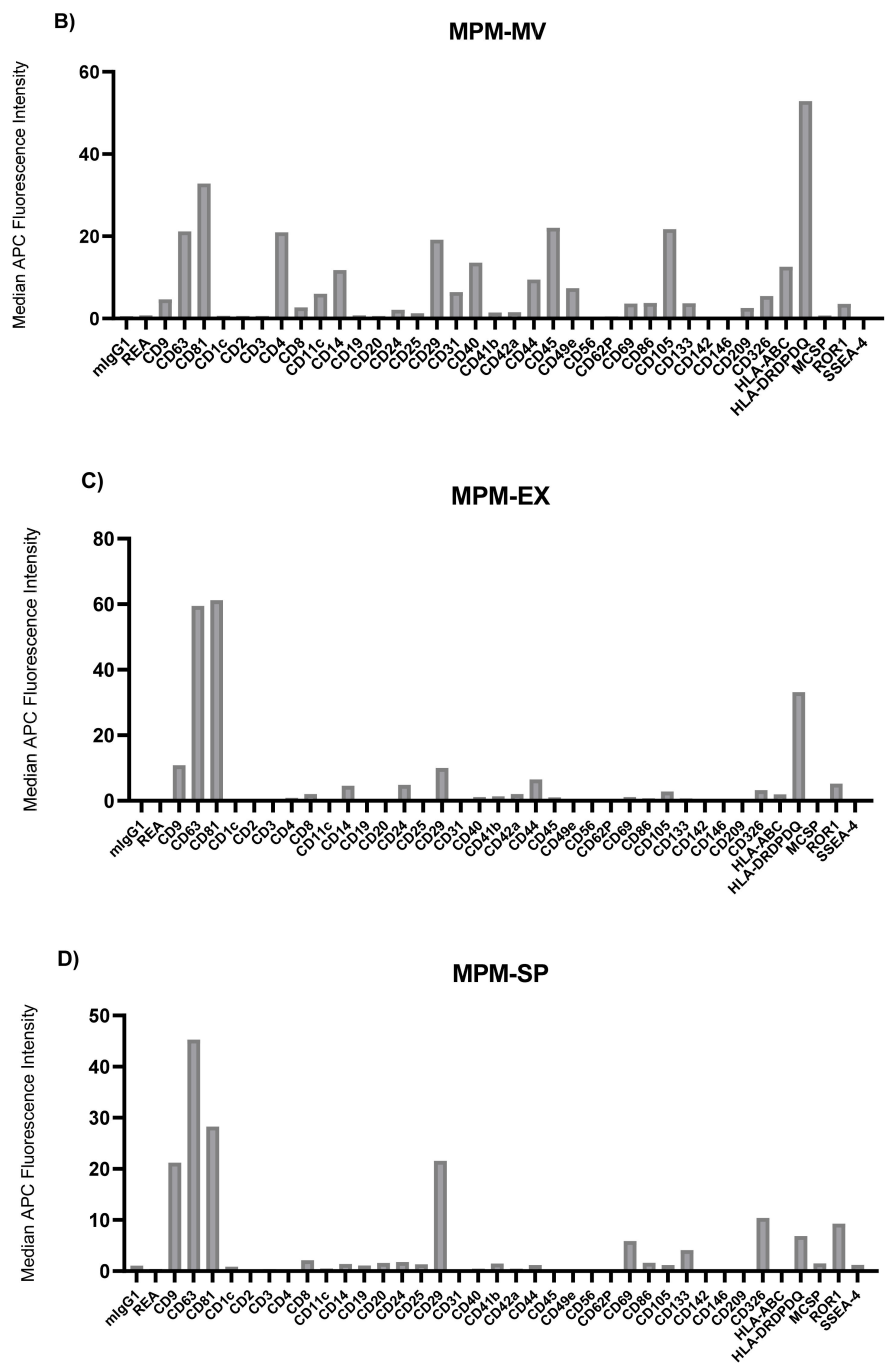
Median fluorescence intensity EV surface protein profiles of malignant pleural mesothelioma (MPM) effusion differential centrifugation fractions: apoptotic bodies (AB) (**A**), microvesicles (MV) (**B**), exosomes (EX) (**C**) and soluble protein in supernatant (SP) (**D**). mIgG1 and REA indicate isotype control and represent negative markers. Each bar corresponds to one surface epitope measurement, as indicated on the x-axis.

To characterize the surface markers present on EVs within the different fractions derived from MPM, AD, and benign patient pleural effusions, we performed a multiplex bead-based flow cytometry assay which was extensively optimized previously [30]. When comparing EV surface protein profiles derived from the different EV subtypes to one another, the highest median fluorescence intensity values were consistently found in the exosome fraction, with the tetraspanins CD63 and CD81 showing the greatest levels. In general, the ratio of CD9 to CD81, as well as the levels of all non-tetraspanin markers decreased during differential centrifugation (Figure 3, Figure 4 and Figure 5). Although there were still significant CD9, CD63, and CD81 signals in terms of their abundance on EVs, this was not as high as implied by our Western blotting results (Figure 1, Figure 3D, Figure 4D and Figure 5D).

There were also large differences between the EV surface profiles detected in benign, AD, and MPM pleural effusions. The most notable trend was that the benign effusion was much less complex than those of AD or MPM, such that only the classic EV tetraspanins, CD9, CD63, CD81, as well as HLA-DRDPDQ, were detected above MFI 15 (Figure 3). The AD effusion showed the next most complex profile in addition to the highest absolute levels of EV tetraspanins, and particularly CD9, of any sample. In addition to these, this sample showed very high levels of CD29 and CD326. Although lower, the AD effusion also contained CD14, CD42a, CD44, CD49e, HLA-ABC, and HLA-DRDPDQ above MFI 5 in multiple EV populations (Figure 4). By direct comparison, MPM effusion contained all of the proteins expressed in AD, except CD42a, in addition to CD4, CD40, CD45, and CD105 (Figure 5). Thus, the MPM effusion was the most complex of the samples studied, though it showed the lowest levels of the EV tetraspanins.

### 3.4. Quantification of Biomarkers in Different Pleural Effusion Fractions

**Figure 6:**
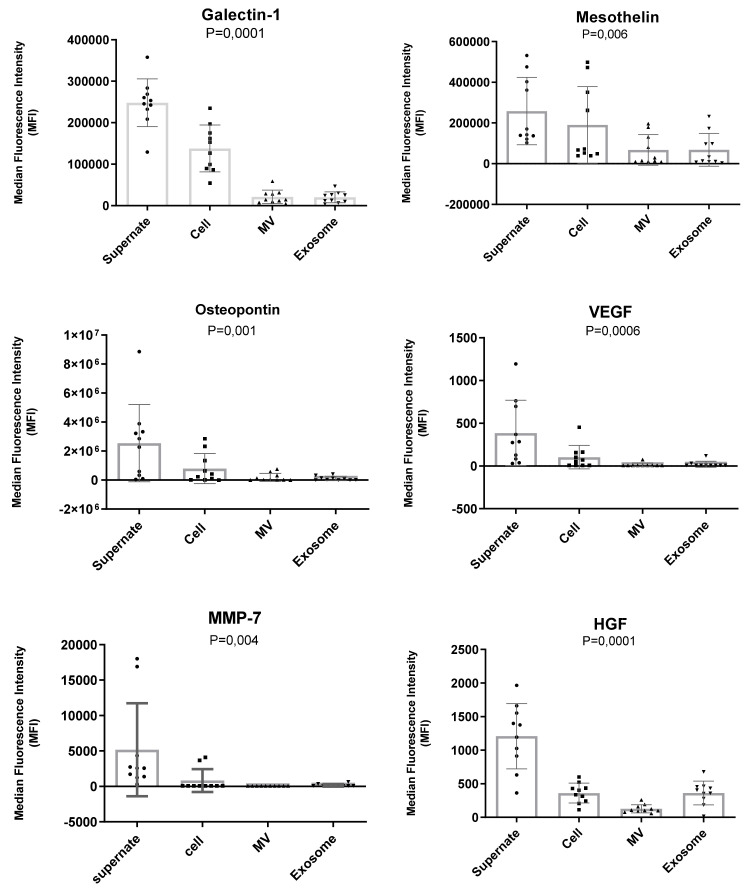

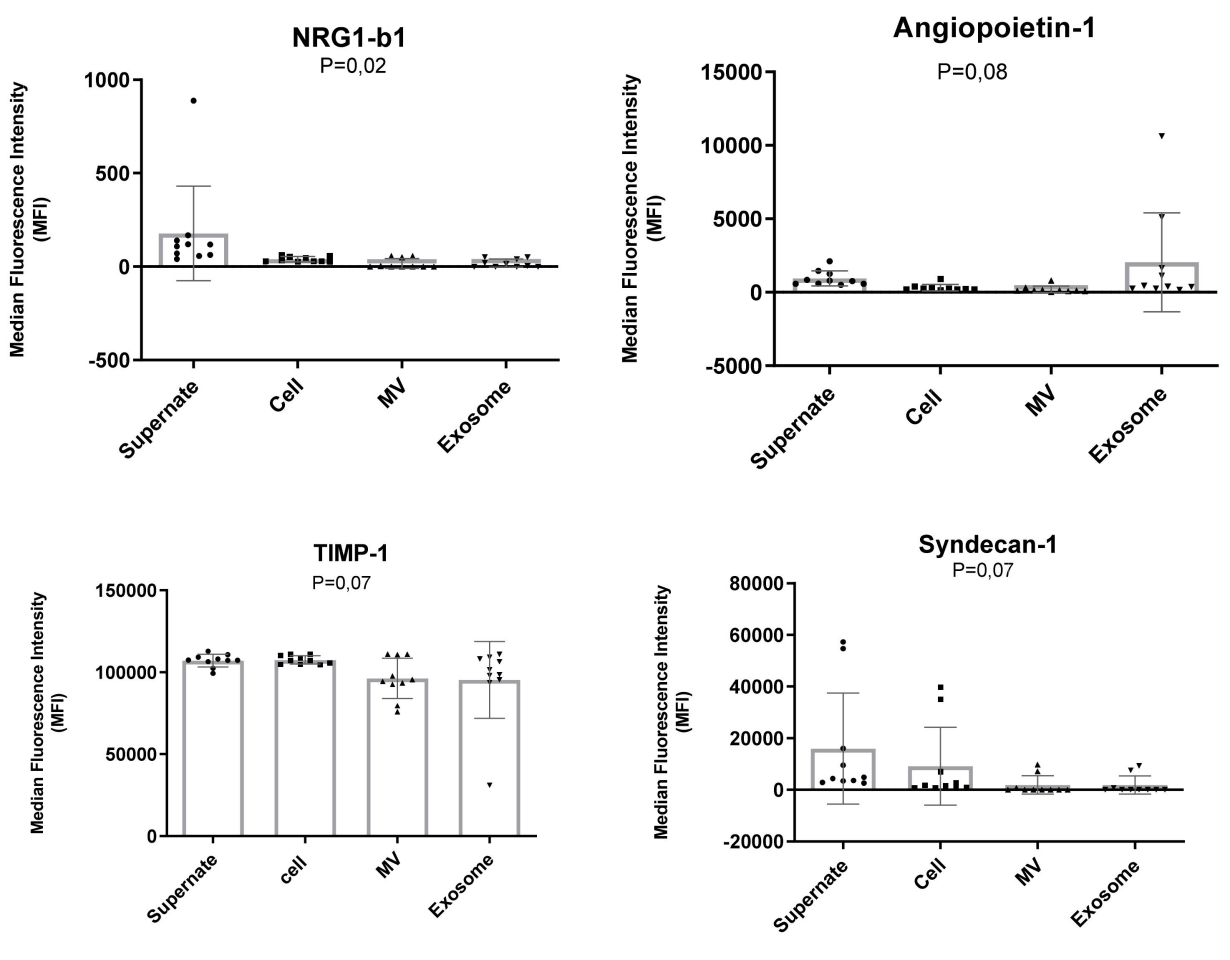
Level of angiogenesis proteins in differential centrifugation fractions of pleural effusion. Levels of Galectin-1, Mesothelin, Osteopontin, VEGF, MMP-7 and HGF were significantly higher in soluble protein when compared with their levels in exosomes. Significance was assessed by two-tailed *t*-test at *p* ≤ 0.05.

Our previous study showed the diagnostic and prognostic value of 10 different angiogenesis proteins in pleural effusion from MPM, AD, and benign patients. Using Luminex assays, we checked the expression levels of these angiogenesis proteins in microvesicles and exosomes derived from pleural effusion from these three patient groups. We detected that the expression level of Galectin-1, Mesothelin, Osteopontin, VEGF, MMP-7, and HGF were significantly lower in exosomes compared with the supernatant of MPM, AD, and benign conditions. Additionally, there were no significant differences in the expression levels of NRG1-β1, Angiopoietin-1, TIMP-1, and Syndecan-1 in the exosomes compared to supernatant (Figure 6).

### 3.5. Presence of Proteins in Exosomes vs. Supernatant 

Next, we focused on whether angiogenesis proteins show different vesicular associations dependent on the disease studied. The results showed that Angiopoietin-1 and TIMP-1 are preferentially transported in exosomes, whereas other proteins occur mainly dissolved in the supernatant with very little in vesicular structures. Osteopontin, Galectin-1, Mesothelin, and VEGF were higher, whereas HGF and SDC-1 were lower in exosomes derived from MPM patients compared to AD and benign patients (Table 1). Interestingly, SDC-1 was represented more in exosomes from AD effusions compared to MPM, while VEGF was represented more in exosomes derived from MPM effusion compared to that from AD patients.

## 4. Discussion

Malignant mesothelioma is an aggressive malignancy with limited therapeutic options. In this research project, we studied exosomes isolated from fresh pleural effusion from patients with malignant pleural mesothelioma, metastatic adenocarcinoma, and benign mesothelial proliferations. 

Pleural effusion contains a variety of cells among them mesothelial cells, macrophages, lymphocytes, leukocytes, as well as malignant cells when caused by cancer. Cell-to cell communication between malignant cells and malignant to non-malignant cells in effusion may play critical role for cancer progression. 

We show that the obtained size and concentration of the exosomes were in accordance with previous studies [32,33,34]. There were differences in their total concentration. The concentrations of exosomes varied among individuals. In the malignant mesothelioma group, three of the effusions came from the same patient but at different time points. The first effusion showed a higher exosomes concentration, and in the subsequent effusions the amount of the exosomes gradually declined. This can depend on many factors, including tumor burden in the pleural cavity and the rate at which vesicles are formed and the fluid volume is replaced after thoracocentesis. The cellular origin of the extracellular vesicles, including the exosomes, can be identified by the surface proteins that reflect the membrane proteins in the original cells [9]. In this study, we demonstrated that the presence of 15 surface proteins (CD9, CD63, CD81, CD2, CD8, CD14, CD29, CD44, CD49e, CD62p, CD105, CD146, CD326, HLA-ABC, and MCSP) were higher, whereas HLA-DRDPDQ and ROR1 were lower on the exosome fraction derived from metastatic adenocarcinoma patient compared to MPM patient suggesting that these markers can be associated with MPM (Table S1). 

These extracellular vesicles carried molecules that regulate a variety of cellular processes, including cell–cell adhesion (CD2, CD8, CD9, and CD146), immune regulation (CD24, CD40), extracellular matrix regulation (CD44), TGF-β receptor activation (CD105), as well as cell growth, differentiation, and migration. The results indicate that all cells present communicate with the pleura as vesicles. The content of these vesicles thus reflects the different regulatory signals present in the effusion.

Additionally, we performed Luminex analysis on differential centrifugation fractions including, apoptotic bodies, microvesicles, exosomes, and supernatant, to measure the concentration of 10 different proteins (among which some were angiogenic-related proteins). In our analysis of extracellular vesicular proteins, Osteopontin, Galectin-1, Mesothelin, and VEGF had higher concentrations in exosomes isolated from MPM patients. In our previous study, we showed that Galectin-1, Mesothelin, Osteopontin, and VEGF have higher levels in malignant pleural mesothelioma compared with the benign patients and Galectin-1 and Mesothelin have higher levels in malignant pleural mesothelioma compared with the metastatic adenocarcinoma patients [29]. These results may explain the higher level of Osteopontin, Galectin-1, Mesothelin, and VEGF in exosomes derived from pleural effusion from MPM patients. 

These proteins are biologically active and can regulate signaling pathways in the recipient cells which leads to alteration of their phenotype [35]. For example, mesothelin can inhibit apoptosis by activating PI3K/Akt and MAPK/ERK signaling pathways or increase cell proliferation by activating the Stat3 signaling pathway. The interaction of mesothelin with CA125 (cell surface glycoprotein) facilitates tumor invasion and metastasis. Osteopontin, Galectin-1, and VEGF regulate many cellular processes, including cell proliferation, adhesion, tumor formation, migration, and angiogenesis [36,37,38,39,40]. 

In addition to this, we showed that SDC-1 and HGF had higher concentrations in exosomes derived from pleural effusion from metastatic adenocarcinoma patients. Exosomes are rich in proteins and previous studies have shown that enzymatic activities and proteins regulate exosomes secretion and mechanisms of proteins loading in exosomes. High heparanase expression influences exosome secretion and regulates their protein contents, among others. SDC-1 and VEGF increase its concentration in exosomes upon heparanase overexpression [41].

The presence of these biologically active proteins may be more stable in exosomes and can actively participate in cell–cell communication by transferring their cargo to recipient cells, eliciting effects related to angiogenesis and therapy resistance. This makes exosomes suitable candidates for future targeting and carriers of precise drug delivery systems. In case these biologically active proteins will be objects for future targeted therapies, it may be important to clarify their possible presence inside vesicles. 

## 5. Conclusions

Exosomes derived from pleural effusion from different patient groups represent different proteins according to their different cell types. Exosomes isolated from pleural effusion from MPM patients have lower levels of CD9, CD63, CD81, CD2, CD8, CD14, CD29, CD44, CD49e, CD62p, CD105, CD146, CD326, HLA-ABC, and MCSP and higher levels of HLA-DRDPDQ and ROR1 surface proteins compared to AD patients. Additionally, Galectin-1, Mesothelin, Osteopontin, and VEGF have higher levels, whereas Angiopoitien-1 has a lower level in exosomes derived from MPM patients compared to the benign patients. Therefore, these proteins can be diagnostic markers for MPM patients.

## Figures and Tables

**Table 1:** Comparison of protein concentration in exosomes and supernatant of specific diseases.

	**Angiopoietin-1**		**HGF**		**Osteopontin**		**TIMP-1**		**Galectin-1**	
Patient Group	Exosomes	Supernatant	Exosomes	Supernatant	Exosomes	Supernatant	Exosomes	Supernatant	Exosomes	Supernatant
AD	49.60%	50.40%	32.50%	67.50%	1.70%	98.30%	48.20%	51.80%	6.70%	93.30%
MPM	33.50%	66.50%	17.20%	82.80%	5.50%	94.50%	49.50%	50.50%	9.30%	90.70%
Benign	81%	19%	32.60%	67.40%	2.10%	97.90%	41.90%	58.10%	5.10%	94.90%
	**Mesothelin**		**NRG1-b1**		**SDC-1**		**VEGF**		**MMP-7**	
Patient Group	Exosomes	Supernatant	Exosomes	Supernatant	Exosomes	Supernatant	Exosomes	Supernatant	Exosomes	Supernatant
AD	7.50%	92.50%	6.50%	93.50%	12.20%	87.80%	0.00%	100%	2.90%	96.90%
MPM	25.40%	74.60%	10.50%	89.50%	0.00%	100%	12.30%	87.70%	2.90%	93.10%
Benign	10.30%	89.70%	8.10%	91.90%	0.00%	100%	0.00%	100%	8.90%	91%

## Supplementary Materials

The following are available online at https://www.mdpi.com/article/10.3390/biom11111606/s1, Figure S1: The diagnostic picture in MPM (a–e), lung adenocarcinoma (f–j) and reactive, but benign, mesothelium (k–n). Immunohistochemical stainings used are Papanicolaou (a,f,k), May Grünewald Giemsa (b,g,l), Calretinin (red)/BerEp4 (brown) (c,h,m), EMA (red)/Desmin (brown) (d,i,n), Mesothelin (e) and TFF-1 (j). In addition to the tumor cells there is benign mesothelium, white blood cells and plenty of macrophages. The mor tumor specific markers (d,e,i,j) show that the tumor cells only make out part of the cell populations. Diagnoses are based on standardized immunocytochemical reaction patterns [42], Table S1: Median Fluorescence Intensity (MFI) values of EV’s surface proteins.
