# Supplementary material for: Diagnostic and Prognostic Utility of the Extracellular Vesicles Subpopulations Present in Pleural Effusion
Source: Biomolecules. 2021 Oct 29;11(11):1606. doi: 10.3390/biom11111606 (PMC8615485; doi:10.3390/biom11111606)
Supplement: Supplementary file 1 [file biomolecules-11-01606-s001.zip › biomolecules-1393944-supplementary.pdf]

Diagnostic and prognostic utility of the extracellular vesicles subpopulations present in pleural effusion

Joman Javadi [1,*], Andre Görgens [2], Hanna Vanky [1], Dhanu Gupta [2], Anders Hjerpe [1], Samir EL-Andaloussi [2], Daniel Hagey [2,+], and Katalin Dobra [1,+]

1. Karolinska Institutet, Department of Laboratory Medicine, Division of Pathology, Stockholm, Sweden; joman.javadi@ki.se; hanna.hjerpe.vanky@stud.ki.se; anders.hjerpe@ki.se; Katalin.dobra@ki.se

2. Karolinska Institutet, Department of Laboratory Medicine, Division of BCM, Stockholm, Sweden; an-dre.görgens@ki.se; dhanu.gupta@ki.se; Samir.el-andaloussi@ki.se; daniel.hagey@ki.se

\+ Shared last authors

* Correspondence: joman.javadi@ki.se; +46-762615122

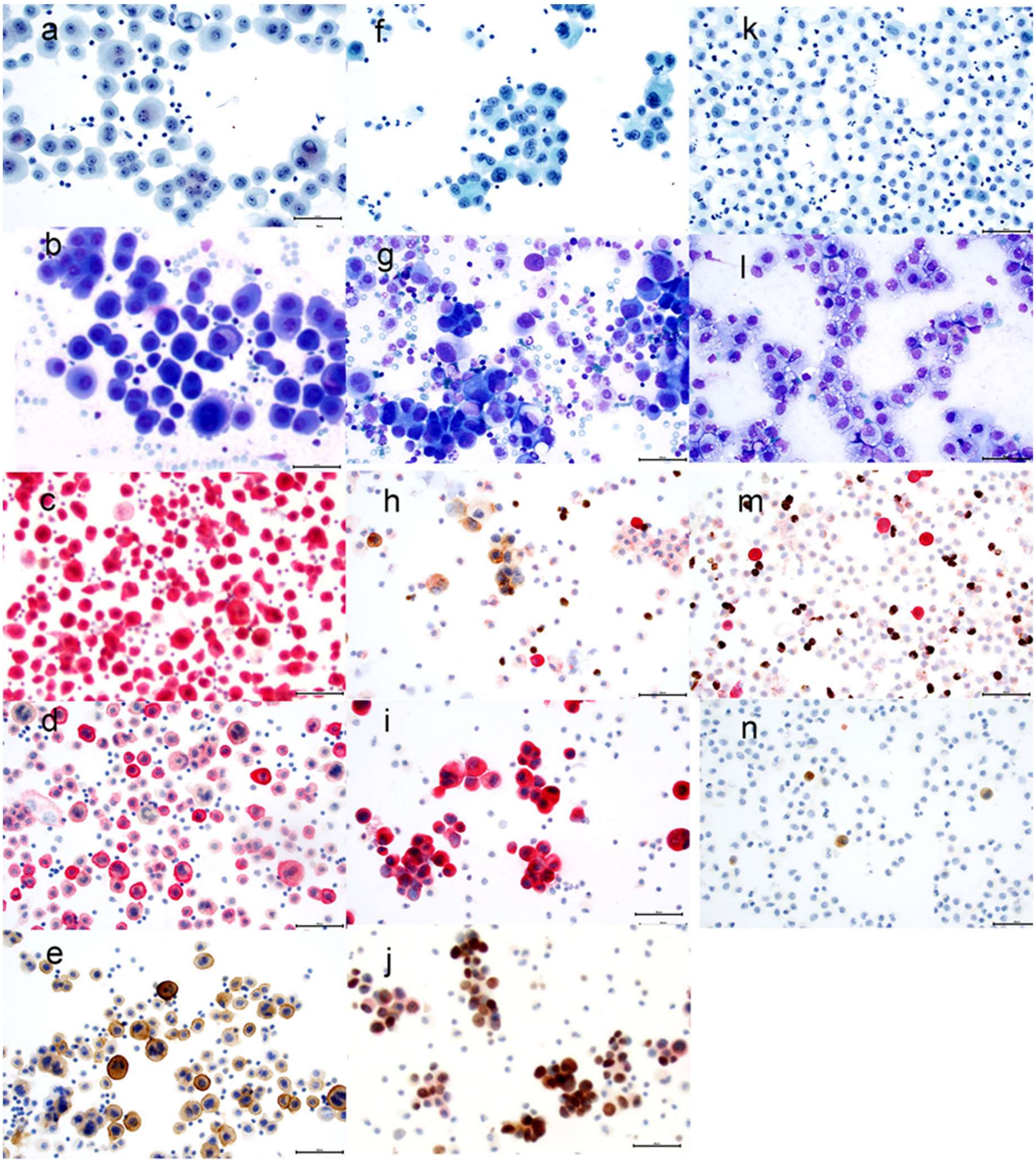

Figure S1. The diagnostic picture in MPM (a-e), lung adenocarcinoma (f-j) and reactive, but benign, mesothelium (k-n). Immunohistochemical stainings used are Papanicolaou (a, f, k), May Grünewald Giemsa (b, g, l), Calretinin (red)/BerEp4 (brown) (c, h, m), EMA (red)/Desmin (brown) (d, i, n), Mesothelin (e) and TFF-1 (j). In addition to the tumor cells there is benign mesothelium, white blood cells and plenty of macrophages. The mor tumor specific markers (d, e, i, j) show that the tumor cells only make out part of the cell populations. Diagnoses are based on standardized immunocytochemical reaction patterns [42].

	CD9	CD63	CD81	CD2	CD8	CD14	CD24	CD29	CD44	CD49e	CD62P	CD105	CD146	CD326	HLA-ABC	HLA-DRDI	MCSP	ROR1
MPM-EX	10,8121	59,5266	61,2166	0,4728	2,0360	4,5797	4,8196	10,0602	6,4972	0,3526	0,3783	2,8229	0,2923	3,2353	1,9596	33,1571	0,3394	5,2184
AD-EX	55,9047	148,1889	146,2502	3,9852	8,2513	25,6659	4,3844	94,5562	16,0027	6,1957	4,7390	6,9342	5,6320	98,4508	5,5771	25,9446	2,8469	1,0913
BE-EX	16,4946	97,8553	100,1408	3,9565	9,9428	5,3813	8,0763	9,3058	10,1203	0,2016	0,3941	2,3153	1,0116	1,2315	0,8816	27,7757	0,0718	5,7566

Table S1. Median Fluorescence Intensity (MFI) values of EV’s surface proteins

Reference

42. Husain, A.N.; Colby, T.V.; Ordóñez, N.G.; Allen, T.C.; Attanoos, R.L.; Beasley, M.B.; Butnor, K.J.; Chirieac, L.R.; Churg, A.M.; Dacic, S. et al., Guidelines for Pathologic Diagnosis of Malignant Mesothelioma 2017 Update of the Consensus Statement From the International Mesothelioma Interest Group. *Arch. Pathol. Lab. Med.* 2018. *142*, 89–108.
